# Against a singular understanding of legal capacity: Criminal responsibility and the Convention on the Rights of Persons with Disabilities

**DOI:** 10.1016/j.ijlp.2015.04.002

**Published:** 2015

**Authors:** Jillian Craigie

**Affiliations:** Centre of Medical Law and Ethics, Dickson Poon School of Law, King's College London, Strand, London WC2R 2LS, United Kingdom

**Keywords:** United Nations Convention on the Rights of Persons with Disabilities, Legal capacity, Criminal responsibility, Autonomy, Value neutrality

## Abstract

The United Nations Convention on the Rights of Persons with Disabilities (CRPD) is being used to argue for wider recognition of the legal capacity of people with mental disabilities. This raises a question about the implications of the Convention for attributions of criminal responsibility. The present paper works towards an answer by analysing the relationship between legal capacity in relation to personal decisions and criminal acts. Its central argument is that because moral and political considerations play an essential role in setting the relevant standards, legal capacity in the context of personal decisions and criminal acts should not be thought of as two sides of the same coin. The implications of particular moral or political norms are likely to be different in these two legal contexts, and this may justify asymmetries in the relevant standards for legal capacity. However, the analysis highlights a fundamental question about how much weight moral or political considerations should be given in setting these standards, and this is used to frame a challenge to those calling for significantly wider recognition of the legal capacity of people with mental disabilities on the basis of the Convention.

## 1 Introduction

Developments in international law in connection with the United Nations Convention on the Rights of Persons with Disabilities (CRPD) have called for wider recognition of the legal capacity of people with mental disabilities. Standard interpretations of the concept “legal capacity” hold that it refers to being a duty bearer as well as a rights holder, encompassing recognition before the law in a broad sense. In keeping with this interpretation, the call for wider legal recognition has been said to apply not only in the context of personal decisions but also in the context of responsibility for criminal acts. This line of thinking assumes that these two kinds of legal capacity are two sides of the same coin: that a need for wider recognition in the personal sphere automatically means that there is a need for wider recognition in the criminal sphere.

It is argued here that this assumption should be resisted. The essential role played by moral and political norms in shaping the relevant standards provides a reason against thinking of legal capacity as a single attribution across legal contexts. Particular moral or political considerations may have different implications in different parts of the law, and this seems likely to justify asymmetries in standards for legal capacity across one legal system. In establishing the implications of the Convention for criminal responsibility one crucial question therefore concerns the implications of the evaluative commitments underlying the CRPD in this particular legal context.

A further, more fundamental question concerns how much of a role moral and political norms should be given in setting any standard for legal capacity. The relevance of such considerations for questions of legal capacity is an underlying theme that runs through the paper. It is used, ultimately, to frame a challenge to those endorsing “strong” interpretations of the Convention, which call for significantly wider recognition of the legal capacity of people with mental disabilities.

## 2 Article 12 and legal capacity

Recent developments in international human rights law have called into question the legitimacy of the current link between mental and legal capacity.^1^ At the centre of these developments, Article 12 of the CRPD requires that legal capacity should not be limited on the basis of mental disability: persons with mental disabilities, including mental disorders, must be recognized as persons before the law on an equal basis to others and must be supported in the exercise of their legal capacity.^2^

The crucial aspect of Article 12, where much of the academic discussion so far has focused, concerns legal capacity in the sense of the right to make one's own personal decisions. While Article 12 has been interpreted in stronger and weaker ways, it is generally understood that the Convention requires states to recognize the legal capacity of people with mental disabilities more widely than is currently the case.^3^ Strong interpretations suggest that Article 12 leaves very limited room for restricting legal capacity on the basis of mental incapacity.^4^ Michael Bach and Lana Kerzner have proposed that the mental requirement for the right to self-determination in one's private affairs should be an ability to express an intention (Bach, 2009; Bach & Kerzner, 2010).^5^ Weaker interpretations hold that the CRPD requires a shift to at least some extent from substituted to supported decisions (Richardson, 2012; Slobogin, 2015).^6^ Therefore, to a lesser or greater degree most interpretations understand the Convention as requiring a lowering of current mental thresholds for legal capacity in the personal sphere, to enable this shift to occur.^7^

However, such calls have been understood to also apply in criminal law, raising a question about the implications of the CRPD for defences that are based on mental incapacities.^8^ Criminal law operates on a presumption that the capacity for crime is present, but this can be displaced if the defendant is under a certain age; or if they meet the requirements of a mental incapacity based-defence (Loughnan, 2011; Peay, 2011a, p. 3). The broad thrust of interpretations of Article 12 can be characterized as the idea that, like everyone else, people with mental disabilities should be free to make their own mistakes, which should be recognized as such; and this idea could plausibly be applied to questions of criminal responsibility.^9^ In 2009 the United Nations High Commissioner reported that the CRPD requires replacing criminal defences that are based on “mental or intellectual disability” with “disability-neutral” doctrines (2009, para. 47), and this has been taken to indicate that defences such as insanity and diminished responsibility may be in violation of the Convention (Bartlett, 2012; Flynn & Arstein-Kerslake, 2014; Slobogin, 2015).^10^

The central aim of this paper is to address a theoretical question about the relationship between legal capacity in these two contexts, in order to address a practical question about the implications of the CRPD for law concerning criminal responsibility. The paper begins by outlining the moral arguments that are given within the CRPD literature, for wider recognition of legal capacity in the personal sphere; and considering whether these *prima facie* apply in questions of criminal responsibility. The arguments present in the literature roughly fall into three categories that I will refer to as “personhood”, “growth and flourishing” and “limited understanding” arguments, which will be discussed in turn.

## 3 Personhood arguments

Prominent among the reasons that are given for wider recognition of legal capacity in the personal sphere is the idea that restricting legal capacity on the basis of mental incapacity involves a failure to properly recognize the individual as a human being (Flynn & Arstein-Kerslake, 2014).^11^ It is argued that people with mental disabilities must be seen as moral subjects rather than as objects to be cared for—that they should be attributed full moral agency rather than being seen as passive recipients of care.^12^ And the importance of this kind of recognition is often justified with reference to the idea that this is essential for meaningful participation in society.^13^

Independent of the CRPD, Martha Nussbaum has made the case for there being a vital link between civil rights and the recognition of people with mental disabilities as citizens with equal human dignity (Nussbaum, 2009). However, as Nussbaum points out, different civil rights seem connected to the notion of equal human dignity to varying degrees. Where there is a close connection Nussbaum argues that the recognition of equal human dignity can only be achieved through an equal capability.^14^ For example, to “give some groups of people unequal voting rights, or unequal religious liberty, is to set them up in a position of subordination and indignity vis-à-vis others. It is to fail to recognize their equal human dignity.” (Nussbaum, 2009, p. 336).^15^ In other domains she argues that it seems that equal human dignity is recognized so long as the relevant capability is “adequately secured” (Nussbaum, 2009, p. 336). Here Nussbaum gives the example of housing, holding that the notion of equal human dignity does not seem to require equal house size beyond a particular threshold. She proposes that domains such as education and basic health care pose more challenging questions about when inequality undermines recognition of equal human dignity, suggesting that in these areas, “adequacy does appear to require something close to [equal access]” (Nussbaum, 2009, p. 337).^16^

Understood against this theoretical background, the personhood arguments in connection with CRPD raise a question about the relationship between self-determination in one's personal affairs, and the recognition of equal human dignity.^17^ Like voting, the liberty to make one's own decisions surely has an intimate connection with respect for equal human dignity. But unlike voting, personal decisions can directly undermine other capabilities that are also connected to human dignity. In this way, it might be argued that the recognition of equal human dignity may sometimes require limits on the right to self-determination.^18^ Nonetheless, Nussbaum's theoretical framework provides one way of grounding and exploring the personhood arguments for greater recognition of legal capacity in the personal sphere.^19^

The question to be considered here is could these personhood arguments equally be made in relation to criminal responsibility? Tina Minkowitz suggests such an argument when she claims that mental disability-based criminal defences involve seeing “persons with disabilities, particularly psychosocial disabilities … as less than moral equals with other persons.” (Minkowitz, 2014, p. 3) And philosophical accounts of what it means to withhold responsibility on such grounds suggest that this may be true at least according to prominent contemporary views. Such accounts offer explanations of what properties of persons make them appropriate targets for blame, and one increasingly influential view holds that a person is blameworthy if their action, or inaction, reflects their rational judgments or normative commitments (Lacey, 2010; Morse, 1999; Smith, 2008; Watson, 1996). Understood in this way, the attribution of responsibility regards the person as answerable for what they have done.^20^

On the basis of one such view, Angela Smith has argued that withholding responsibility is “in effect, to say that [the person] is not to be regarded as someone to be reasoned with, but merely as someone to be understood, treated, managed, or controlled. It is to regard a person as we would regard a vicious dog or a bratty toddler … not someone with whom it is possible to enter into relationships of mutual respect and recognition” (Smith, 2008, p. 389).^21^ On the more narrow issue of withholding criminal responsibility, Antony Duff holds that this entails that the individual is incapable of reasoning to the degree that “we cannot address them as fellow participants” in the relevant legal practices (Duff, 2005, p. 450).

A similar understanding of what it means to withhold criminal responsibility is found in discussions of specific defences. John Gardner has explored the meaning of the defence of diminished responsibility in his reflections on why, intuitively, someone who suffers domestic abuse and kills their abuser should prefer a finding of provocation to diminished responsibility, despite the fact that both would reduce a charge of murder to manslaughter. He suggests that by making use of the defence of diminished responsibility the defendant opts for a non-rational explanation of the behaviour and thereby “demeans herself as a rational being”. In his view, a successful defence amounts to seeing the person as “someone who can't explain herself rationally, someone … whose participation in the human good is diminished” (Gardner, 2007, pp. 180–181).^22^

Such accounts suggest that withholding criminal responsibility on the basis of mental incapacity does involve a particular kind of social exclusion, and at least a partial withdrawal of the recognition that we normally extend to others. On this basis, the personhood arguments that are given as a reason for wider recognition of legal capacity in the personal sphere do seem relevant to incapacity-based criminal defences. In this way, a case could be made that defences such as insanity and diminished responsibility do undermine the recognition of personhood, and that this provides a reason to adopt minimal mental requirements for criminal responsibility.^23^

## 4 Personal growth and flourishing arguments

A second set of arguments for wider legal recognition of people with mental disabilities in the personal sphere more directly concerns consequences. According to these arguments, allowing people with mental disabilities to make their own decisions will enable their personal growth and the development of capacities for flourishing (Bach, 2009; Flynn & Arstein-Kerslake, 2014; Quinn, 2010).^24^ These are empirical claims, and they will remain speculative until the policies in question are put in place. Nevertheless, it seems a plausible hypothesis that many people with mental disabilities could be helped to develop and flourish through supported rather than substituted decisions (as a result of lowering current mental thresholds for legal capacity in the personal sphere).^25^ The developmental importance of making one's own choices is widely recognized, an obvious example being the role that self-determination is thought to play in development of personal autonomy through childhood and early adulthood. Common sense says that young people must be allowed to exercise their developing agency, exposing them, necessarily, to certain risks involved.^26^

Transferred to the sphere of criminal law, the idea would be that assigning criminal responsibility also plays a role in personal development. Duff suggests that it is unlikely that criminal law has a single purpose (Duff, 2011). However, censure is a prominent function of criminal justice systems, and perhaps in connection with this element considerations of personal development are found in English and American law at least as far back as the early 19th century.^27^ At that time a strict imposing of liability was understood to serve a character building function, “teaching deficient individuals how to become responsible subjects” (Blumenthal, 2007, p. 1158). A similar perspective is found today in the justifications sometimes given for holding children criminally responsible (for discussion see: Keating, 2007).

The crucial issue for the purpose of this paper is whether these ideas about what the assigning of criminal responsibility might achieve for someone with a mental disability, will be borne out. In practice, within current legal systems the question is which of two paths is most likely to promote this developmental end: punishment, possibly prison, and a criminal record; or diversion out of the criminal justice system, and treatment, possibly involving a hospital disposal? It would seem that the answer depends enormously on the resources that are available along both paths. However, my tentative hypothesis is that we should be sceptical about there being a greater opportunity for growth and development through the criminal justice system; more sceptical, at least, than about the possibilities for growth and development through recognizing legal capacity in the context of personal decisions.^28^ One reason for skepticism is there being fewer opportunities for supported decision-making in this context given that many crimes are spontaneous.^29^

These are ultimately questions that await further investigation. However, at least in the context of existing Anglo-American jurisdictions, arguments based on personal growth and flourishing as an outcome of recognizing legal capacity transfer less well to the sphere of criminal responsibility than personhood arguments. This identifies one way that a proponent of wider recognition of legal capacity in the context of personal decisions might resist the corollary that an equivalent approach should be adopted in questions of criminal responsibility. The claim would be that while personhood arguments may provide a reason to pursue greater recognition in this context, personal growth arguments do not. The question then becomes one about the relative importance of the “personhood” and “personal growth” considerations.

## 5 Limited understanding arguments

A third kind of argument present in the literature is based on developments in the understanding of human decision-making, and a claim about how limited this understanding still is. Eilionoir Flynn and Anna Arstein-Kerslake point to recent developments in psychology that have emphasized the importance of emotional and intuitive mental processes in decision-making, which in their view call standard functional tests into question because of their focus on “cognition and rationality” (Flynn & Arstein-Kerslake, 2014, p. 82).^30^ Flynn and Arstein-Kerslake argue that given these developments and the likelihood that we still have much to learn, in the sphere of personal decisions the law should err on the side of respecting legal capacity. The error that we should try to avoid is denying legal capacity when someone may in fact be in a position to make their own choices, and this is said to provide a reason for wider recognition of legal capacity in this context.

The arguments concerning what the scientific developments mean for current legal capacity standards are yet to be fully articulated and explored,^31^ but it is clear that our understanding of human decision-making is currently limited. This concern straightforwardly applies to current functional tests in any legal context. However, different conclusions may well be drawn about what our response should be in the context of different legal questions. In law concerning personal decisions, a choice must be made between prioritizing liberty by recognizing legal capacity, or protecting well-being. But in law concerning criminal responsibility there is a different choice to be made. In this context, by erring on the side of recognizing legal capacity one avoids the risk of excusing someone and diverting them out of the criminal justice system (or holding them responsible for a less serious offence) when they should in fact be held fully responsible. The risk that is preferred is the punishment of people who should be diverted out of the criminal justice system.^32^ Whether this seems like the right risk to choose rests on a judgement about the relative seriousness of these outcomes.

What becomes apparent is that this third kind of argument depends on moral considerations such as those identified in the previous two sections: issues such as the importance of recognition as an agent in the context of criminal acts, and the likelihood and importance of promoting personal development through the assigning of responsibility. On its own, our limited understanding of human decision-making does not adjudicate either way on the question of whether there should be wider recognition of legal capacity—either in relation to personal decisions or criminal acts.

## 6 Interim conclusions

The foregoing overview of the moral arguments for wider recognition of legal capacity present in literature connected to the CRPD suggests that the three kinds of argument identified apply to questions of criminal responsibility to varying degrees. Personhood considerations arguably provide a reason to endorse minimal mental requirements for criminal responsibility. However, the implications of arguments concerning personal growth and flourishing are more uncertain. Proponents hold that wider recognition of legal capacity in the personal sphere will facilitate the personal growth and flourishing of people with mental disabilities, but this seems less plausible as an outcome of greater recognition of legal capacity in questions of criminal responsibility. Among the reasons for this conclusion, there seems to be much less opportunity for supported decision-making in this context. Developmental considerations therefore offer one way that a proponent of wider recognition of legal capacity in the sphere of personal decisions might resist endorsing an equivalent position in relation to standards for legal capacity in the context of criminal acts.

The third kind of argument that was identified concerns the significance of our still limited understanding of human decision-making. The moral question that arises is, given the uncertainty in this area, how should lawmakers respond? It was argued that the answer may well be different in the context of personal decisions and criminal acts, because of the different evaluative issues at stake. While decisions about where to set the mental threshold for the right to make one's own decisions must balance the value of liberty and recognition against the protection of well-being, the equivalent question in relation to criminal responsibility involves weighing the importance of punishment and holding others to account, against welfare considerations. This third category of argument therefore potentially offers another source of reasons against the wider recognition of legal capacity in questions of criminal responsibility.

A broader implication of the above analysis is that it suggests there may be strong moral reasons not to think of legal capacity in a singular, decontextualized way: it may well be appropriate to adopt different standards for legal capacity in different areas of law, on the basis that there are different moral and political considerations in play. This raises a question about why legal capacity is being understood in a singular way in the CRPD literature, such that claims about legal capacity in the context of personal decisions are assumed to automatically apply to questions of legal capacity in the context of criminal acts.

One motivating concern may be about the fairness of one person, at a single point in time, being recognized as having legal capacity in one context but not another.^33^ In relation to the implementation of the CRPD the specific concern would be that adults with mental disabilities might be recognized as legal agents in their personal decisions, but not in relation to criminal acts. One place in English law where there is a clear asymmetry in the assigning of legal capacity between personal decisions and criminal acts, and where concerns about this have been raised, is in law concerning young people. In England and Wales, a person is held fully criminally responsible at age 10, but it is not until age 18 that they gain the full legal capacity to make their own treatment decisions.^34^ The asymmetry here is the converse of the situation just considered in relation to adults with mental disabilities—a 10-year-old is recognized as a full legal agent in relation to criminal responsibility but not treatment choices. Nonetheless, this situation in existing law provides a starting point for considering the general issue of asymmetry.

## 7 An argument for uniformity

In a critique of this state of affairs for young people, Barry Lyons has argued that there should be a strong presumption in favour of uniformity in these attributions of legal capacity (Lyons, 2010).^35^ His argument rests on a parallel that he draws between the mental abilities that are presumed necessary for these two kinds of legal capacity. Broadly in keeping with criminal jurisprudence,^36^ Lyons holds that as a general principle it is, “unfair to ascribe [criminal] responsibility to individuals who were not capable of self government”, and therefore that ascriptions of responsibility assume that the person in question is capable of self-government (Lyons, 2010, p. 273).^37^ He further argues that self-government in this context involves essentially the same mental functions as self-government in the context of personal decisions—an ability to weigh up options with reference to a set of values, and to understand the consequences of choices and actions—and that the decisions in question can be equivalently difficult and complex.^38^

On the basis of these parallels, Lyons recommends either that if “children are to be held accountable by the criminal justice system then it seems that we should recognise their capacity to make their own healthcare decisions”; otherwise, if the law does not allow children to make their own healthcare decisions, “the criminal justice system would seem to have little entitlement to hold them responsible for acts that transgress the criminal law.” (Lyons, 2010, p. 278).^39^ Crucially, Lyons explains the asymmetry in existing law in terms of the pursuit of different ends. According to Lyons, in the medical context the law aims to preserve the lives of young people by denying them the right to refuse life-saving treatment, resulting in a high age threshold for full legal capacity; while in the criminal context a desire to hold children responsible results in a much lower age threshold for legal capacity (Lyons, 2010, pp. 277–278).^40^ In his view, the role played by these considerations constitutes a “manipulation” of the relevant standards (Lyons, 2010, p. 278)—a claim that seems to imply that it is inappropriate for moral or political considerations to play a role in setting these requirements for legal capacity.

What's missing in this picture is that even a complete understanding of the mental functions that are engaged in the relevant decisions and actions would not answer the question of what the legal standards should be. This is a necessarily normative question, in part because for any mental function that is implicated, there remains a question about *how much* functioning is necessary for the recognition of legal capacity in the relevant domain. It is increasingly recognized that autonomy (or self-governance) in this psychological sense comes in degrees, and that we can all be helped to be more autonomous in our personal decisions (Craigie & Bortolotti, 2015; Maclean, 2006; O'Neill, 2002; Thaler & Sunstein, 2009). It seems that there is no natural threshold for the legal standard (for discussion in relation to the Mental Capacity Act 2005 in England and Wales see: Richardson, 2012, 2013). Rather, the boundary between mental capacity and incapacity in the private sphere is drawn in part on the basis of moral and political commitments such as the value of liberty, well-being and life (Buchanan, 2004).^41^ These considerations shape what is considered minimally necessary in terms of mental functioning for the legal capacity to make one's own personal decisions. As a result, different societies, or the one society at different times, will draw this line in different places on the basis of divergent evaluative commitments.

For the same reason it also seems that one society at a single point in time may be justified in adopting different standards for legal capacity in different parts of the law. In relation to law concerning children, this is a possibility that has been raised by Heather Keating. Keating argues that questions of where to set the age thresholds for criminal liability and self-determination are not purely developmental matters—rather, they are policy decisions which are therefore “shaped in part … by political considerations.” (Keating, 2007, p. 191). On this basis, Keating frames the question about whether an asymmetry between these thresholds is justified, as one about whether the political purposes of the two legal standards are sufficiently different to justify the discrepancy.

However, Keating also points to a further question concerning the appropriate *extent* of the role for political considerations in setting these standards. Even if one thinks that the political considerations are very different between these two legal contexts and that they justify an asymmetry, there remains a question about how much of a role any moral or political consideration should play in determining these standards. In Keating's view, the current age threshold for criminal responsibility has been shaped to too great a degree by the punitive aims of the criminal law, while too little consideration has been given to a 10-year-old's psychological limitations. In effect, the balance between these two kinds of consideration—what I will call “evaluative” and “psychological” considerations—is unjust.^42^ Applying this approach to law concerning adults, and adopting the plausible assumption that there is considerable overlap in the psychological functions engaged in personal decisions and criminal acts, only a limited degree of asymmetry between standards for legal capacity in relation to personal decisions and criminal acts will be justified.

This account of the proper relationship between evaluative and psychological factors in questions of legal capacity brings a central feature of the developments around the CRPD into focus. Those adopting strong interpretations of Article 12—calling for much wider recognition of the legal capacity of persons with disabilities—implicitly reject this account, holding instead that evaluative considerations should be given much more weight than psychological considerations. Since the arguments for uniformity are based on psychological considerations, they appear to have little bearing on this kind position. The relationship between evaluative and psychological considerations that is implicit in the strongest interpretations of Article 12 allows for virtually any degree of asymmetry between standards for legal capacity in different parts of the law, so long as the difference is justified in moral or political terms. Moral arguments referring to, for example, personhood or personal development might be still given against a *particular* asymmetry in legal capacity (this possibility is considered in the final section of the paper). However, the asymmetry under consideration here is a person having legal capacity in relation to personal decisions but not criminal acts, and at least intuitively, this appears less morally problematic than the converse situation for children in English law.

## 8 Case study: 19th century American law

The role played by evaluative considerations in setting standards for legal capacity, and tensions between the perceived importance of evaluative and psychological considerations, has a long history that is vividly illustrated in 19th century American law. In a rich historical study, Susanna Blumenthal describes how political and moral commitments played a profound role in determining the mental requirements for legal capacity during this period (Blumenthal, 2007). Blumenthal argues that a concept she calls the “default legal person”—broadly referring to abilities for understanding, rational judgement and the ability to carry out one's own purpose—underpinned attributions of legal capacity throughout American law of the 1800s. However, her analysis shows how social pressures were explicitly acknowledged as factors that could legitimately constrain the shape of the default legal person in particular legal contexts.

Blumenthal describes how at the beginning of the 19th century, notions of mental incapacity played little role in American law—psychological considerations were not considered relevant in questions of legal capacity. The Enlightenment perspective of the time emphasized the individual's ability to overcome mental weakness, and this was combined with a strong concern for the preservation of America's developing financial system.^43^ However, as the 19th century progressed, the burgeoning field of medicine became critical of what was increasingly seen as an overly optimistic picture of human capacities to overcome mental illness. Gradually, a new legal perspective emerged, “cast in liberal humanist terms” which were felt appropriate for an independent America, and mental ability became seen as essential to legal capacity (Blumenthal, 2007, p. 1166).

However, this transition played out in different ways in different parts of the law. Blumenthal details how law concerning civil commitment and guardianship initially saw a shift in the mid-1800s to a focus on the well-being of the individual and those around him, which was buttressed by a growing faith in medicine. These factors raised the mental threshold for legal capacity in this area of law. However, discontent voiced by those emerging from interventions based on this raised threshold—some of whom brought false imprisonment suits and sought damages—turned the tide against this evaluative frame. In the second half of the century the protection of patients' rights became paramount, once again lowering the mental threshold for this kind of legal capacity but this time for very different reasons than were given for the low threshold at the beginning of the century.^44^

In the context of contract law, Blumenthal describes how over the first half of the 19th century the importance of preventing the ruin of people who could not understand the contracts they entered was increasingly emphasized, raising the threshold for competence to contract.^45^ It was judged, however, that the threshold for the capacity to make a will should remain low, and a number of justifications were given for this position. In particular, the low threshold aimed to preserve the testator's motivation to accumulate wealth, and to ensure his family's motivation to care for him when the need came in his later years.^46^

Law concerning torts also began with an extremely “undemanding” standard at the beginning of the 19th century, with the capacity to do harm being sufficient for liability (Blumenthal, 2007, p. 1178). Blumenthal explains that this approach was justified, among other reasons, on grounds that the person who suffered the loss was entitled to compensation. But gradually the moral outlook in this context also shifted to focus on the apparent injustice of holding a person who could not have avoided the injury because of a mental incapacity, to the same standards as a person with full mental powers. By the beginning of the 20th century legal opinion had come around to the idea that blameworthiness was a fundamental requirement of liability, and that this required an opportunity to avoid the harm, which might sometimes be absent due to mental disability.

This historical window describes significant shifts in thinking about legal capacity, from psychological factors playing virtually no role and legal capacity being determined almost entirely by evaluative considerations; to the concept of mental abilities becoming recognized as a relevant factor in questions of legal capacity; and then to various attempts at balancing psychological and evaluative considerations. Against this background, the recent calls for much wider recognition of the legal capacity of people with mental disabilities represent a return to the early 19th century approach according to which psychological considerations had little role to play—this time not on the basis of an Enlightenment faith in reason, or to encourage trust in the financial system, but on grounds of a commitment to the importance of recognizing the personhood and promoting the personal growth and flourishing of people with mental disabilities. With this understanding of these legal developments in hand, the final section of the paper returns to the question of the relationship between standards for legal capacity in the context of personal decisions and criminal acts, in light of the CRPD.

## 9 The CRPD and legal capacity in relation to personal decisions and criminal acts

Understood very broadly, there are four possibilities for the relationship between the two standards in question. Perhaps the least compatible with the new thinking about mental disability in connection with the CRPD is the possibility of equivalent, demanding mental requirements for legal capacity across both contexts. Such a position would allow considerable scope for withholding the recognition of legal capacity on the basis of mental incapacity, both in relation to personal decisions and criminal acts. The evaluative outlook that seems most likely to underpin such a position today is a protective stance in relation to people with mental disabilities, which might be explained in terms of an intrinsic and significant vulnerability. However, this way of understanding mental disabilities is deeply inconsistent with the conceptual and evaluative frame of the CRPD.

A second possibility is that of a more demanding standard (or standards) for criminal responsibility than for the right to make one's own personal decisions.^47^ This combination would allow more scope for withholding blame and punishment on the basis of mental incapacity, recognizing legal capacity more readily in the personal sphere. This is one position that seems compatible with the evaluative commitments found in the CRPD and surrounding literature. As set out earlier, the reasons for such a position could appeal to the idea that the personal development and flourishing of people with mental disabilities is unlikely to be promoted by low mental thresholds for criminal responsibility; or perhaps to the idea that recognizing personhood is much less important in this context. Seemingly in support of this last possibility, the Committee on the Rights of Persons with Disabilities explains that legal capacity “acquires a special significance for persons with disabilities when they have to make fundamental decisions regarding their health, education and work” (2014: para. 8), omitting any reference to criminal responsibility. A further reason that might be given in for this position is the much greater opportunity for decision-making support in the context of personal decisions.

A different kind of justification for this position might appeal to psychological rather than moral or political reasons (though as I have suggested, this kind of argument seems unavailable to those adopting a strong interpretation of Article 12).^48^ It could be argued that acting responsibly in a social context requires mental abilities that go beyond those that are necessary for acting (or deciding) in accordance with one's own will and preferences, and that this provides a reason for adopting a more demanding standard for criminal responsibility (Craigie & Coram, 2013). However, this is an issue on which there seems to be considerable disagreement and it is not clear how it could be resolved. Lyons argues (and Keating seems to agree) that the mental functions engaged in these tasks are essentially the same (Keating, 2007; Lyons, 2010). But it seems equally plausible to adopt the position that understanding the norms of society is in fact a simpler task than developing the self-knowledge that is necessary for pursuing one's own interests.^49^ The norms of society are omnipresent from childhood, while coming to know oneself and developing one's own projects and identity might be considered a much more difficult task. Understood in this way, competent choices in the personal sphere require more mental maturity than acts in accordance with the criminal law.

A third possibility is that of a more minimal standard for criminal responsibility than for legal capacity in the context of personal decisions. This combination would allow for considerable interference in personal decision-making on grounds of mental incapacity, but would make it more difficult to use mental incapacity as a basis for withholding or reducing blame and punishment.^50^ This combination does not seem to fit well with the evaluative commitments found in the CRPD literature, but a number of other moral orientations could be used to justify such a position. In particular, social conservatives are associated with being “tough on crime” but also being committed to substantive views on the value of life and health, which are used to argue for an interventionist stance in the personal sphere. Cast in jurisprudential terms, Lyons considers a similar justification that, “the criminal justice system has a responsibility to prosecute wrongdoing, while it seems the family courts should err on the side of life rather than death.” (Lyons, 2010, p. 275).^51^ The underlying idea is that these two parts of the legal system are constituted by fundamentally different values. Describing this in a study of 19th century English law, Ezra Hasson contrasts what she calls the “humanitarian” values of the civil law with the “social” values of the criminal law (Hasson, 2010, pp. 17–18).

However, an alternative, unifying, explanation for this combination of standards is that a fundamental commitment to the value of human life and well-being—or perhaps a disvaluing of personal harm—pulls in opposite directions in these two legal contexts. In questions of criminal responsibility these commitments mean that a defendant's mental incapacities have to be significant to count as exculpating, particularly in the context of crimes that involve serious personal harms such as a killing. In the context of personal decisions, particularly decisions concerning healthcare, these same values drive the mental threshold for legal capacity in the opposite direction, allowing the state greater scope to preserve life and well-being.

The final broad possibility is that of equivalent, minimal standards in both contexts. This is the second place where a proponent of wider legal recognition for people with mental disabilities might position themselves, though the threshold endorsed will depend on how strongly Article 12 is interpreted. In justifying such a position it might be argued that personhood considerations are much more weighty than personal growth and flourishing considerations; and that personhood considerations offer equally strong reasons for recognizing legal capacity in relation to personal decisions and criminal acts. Alternatively (though much less convincingly in my view), it might be argued that personal growth and flourishing considerations also weigh in favour of wider recognition of legal capacity in relation to criminal acts.

## 10 Conclusions

It has been argued here that the essential role played by evaluative considerations in setting functional standards for legal capacity may justify asymmetry between these standards across one legal system. This account of the role played by evaluative factors rejects the assumption that legal capacity in the context of personal decisions and criminal acts are two sides of the one coin; as well as the corollary that a position adopted in one context must automatically apply to the other.

The evaluative frame endorsed in the literature around the CRPD is compatible with two broad possibilities: a more demanding mental standard (or standards) for criminal responsibility than for the right to make one's own personal decisions; and equivalent, minimal standards in the context of both kinds of legal question. Proponents will no doubt differ in their answers to the various moral questions posed in this paper—Does an incapacity-based criminal defence undermine the recognition of personhood? Is the recognition of personhood equally important in these two legal contexts? Is it likely that assigning criminal responsibility will be developmental for people with mental disabilities? Which should be given more weight: issues of personhood or personal development and flourishing?—And differences in these answers will deliver a different conclusions regarding the implications of the CRPD for law concerning criminal responsibility.

It has been an underlying purpose of this paper to examine the interplay between psychological and evaluative considerations in questions of legal capacity; and to draw attention to how these two types of consideration are being balanced in the developing literature around the CRPD. Strong interpretations of Article 12 radically prioritize evaluative over psychological considerations in questions of legal capacity. The idea that justice limits the role that should be played by evaluative considerations in setting these standards therefore presents a challenge to such views. As lawmakers endeavour to respond to the CRPD, a critical issue that deserves further investigation concerns why, as some proponents claim, this construction of the relationship between evaluative and psychological considerations is the one that should be pursued.

## Footnotes

1 As described by the Committee on the Rights of Persons with Disabilities, “Legal capacity is the ability to hold rights and duties (legal standing) and to exercise these rights and duties (legal agency)” (2014, para. 13).

2 The United Nations Convention on the Rights of Persons with Disabilities was adopted by the UN General Assembly in December 2006 and came into force in May 2008; *Ibid,* para. 25.

3 Peter Bartlett writes that, “To what extent the concept of mental capacity can still be used” is an “open question” in the interpretation of the Convention (Bartlett, 2012, pp. 761–762). Wayne Martin and colleagues argue that the functional test of the Mental Capacity Act 2005 in England and Wales, as written rather than as applied in practice, is compatible with the CRPD. However, they note that this interpretation is explicitly rejected by the CRPD Committee (Martin, Michalowski, Jütten, & Burch, 2014).

4 For example, the CRPD Committee's general comment on Article 12 states: “Article 12 of the Convention affirms that all persons with disabilities have full legal capacity.” (para. 8) “Under Article 12 of the Convention, perceived or actual deficits in mental capacity must not be used as justification for denying legal capacity.” Committee on the Rights of Persons with Disabilities (2014, para. 13). A discussion paper by Lucy Series and colleagues explains that, “The aim of the GC in this area is to ensure that legal capacity is de-coupled from prejudicial perceptions of an individual's ‘mental capacity.’ It seeks to ensure that regardless of an individual's level of decision- making skills, she or he is still respected as a person before the law and a legal agent. If intervention in legal decision-making does occur, it must be based on factors that all individuals could be subject to, not merely people who have a cognitive disability or are perceived as lacking decision-making skills.” (Series, Arstein-Kerslake, Gooding, & Flynn, 2005, p. 5) For a critique of such interpretations see John Dawson's contribution to this journal issue (Dawson, 2015).

5 This is a much less demanding standard than those currently used in many jurisdictions, which require abilities to do with understanding, appreciating, using and weighing relevant information. Commenting on such “functional” tests, Bach and Kerzner write, “To make recognition of legal capacity dependent on a particular set of decision-making skills, as most current capacity assessments do, is to import ableist assumptions about what the demonstration of decision-making ability entails.” (Bach & Kerzner, 2010, p. 66).

6 In this journal issue, Christopher Slobogin discusses his “basic rationality and basic self-regard” test which in his view constitutes a “narrow definition of competency” (Slobogin, 2015, p. XX). However, he recognizes that this may not be considered compatible with the CRPD on certain interpretations. Also in this journal issue, Bernadette McSherry and Kay Wilson argue for a moderate position, calling for a shift of focus from negative rights to positive rights and the provision of support mechanisms: “the CRPD highlights that there may be a midway point between involuntary treatment on the one hand and no treatment at all on the other” (McSherry & Wilson, 2015, p. XX).

7 The exception would be interpretations that resist any shift from substituted to supported decision-making beyond cases where support enables the person with a disability to satisfy an existing mental capacity requirement.

8 Michael Bach writes that having legal capacity includes being “held legally responsible and liable for one's actions in contract, tort, property and criminal law” that is “to be one to whom legal responsibilities attach.” (Bach, 2009, p. 3). A further issue not addressed in this paper concerns unfitness to plead. For an in depth discussion in the context of Australian commonwealth law see: Australian Law Reform Commission (2014). See Jill Peay's contribution to this journal issue for an English Law perspective (Peay, 2015).

9 Throughout the paper I am using the phrase “recognition as a legal agent” to refer to both elements of legal capacity as described by the Committee on the Rights of Persons with Disabilities (2014). See footnote 1.

10 For an examination of this question in the context of English law see: Peay (2015). Peay notes that on issues of mental capacity in criminal law the CRPD and the European Convention on Human Rights (ECHR) are driving in opposing directions. She argues that—apparently mindful of the ECHR—the Law Commission's recent recommendation that mental capacity be adopted as the concept underpinning the insanity defence, would “[arguably offer] more protection from criminal liability to those with a disability; but it would arguably also bring the law into greater conflict with the CRPD.” (p. XX). Also in this journal issue, Alec Buchanan critiques the Law Commission's recommendation for a mental capacity-based insanity defence: (Buchanan, 2015) Bach and Kerzner recommend that, “legal responsibility can only be diminished where a person lacked the requisite decision-making capability when carrying out actions which are now the focus of civil or criminal proceedings. Decision-making capability would then have three main components: a) decision-making abilities that meet the minimum threshold as defined above; b) needed decision-making supports …; and c) reasonable accommodation on the part of others in the decision-making process (i.e. the goods and services).” (Bach & Kerzner, 2010, p. 71). However, Bach elsewhere suggests that incapacity-based criminal defences are not problematic in the way that incapacity-based standards used to override the right to make one's own choices are: “one of the ‘bases’ on which non-disabled ‘others’ enjoy legal capacity, is precisely their right to invoke incapacity, as temporary or as long term as it might be, as a defence in contract, tort and criminal law. It would be difficult to argue that this right to invoke incapacity, as a basis on which ‘non-disabled others’ exercise legal capacity, is a violation of Article 12. Such a defence is foundational to contract, tort and criminal law.” (Bach, 2009, p. 5).

11 From the Committee on the Rights of Persons with Disabilities: “Legal capacity means that all people, including persons with disabilities, have legal standing and legal agency simply by virtue of being human” (2014, para. 12).

12 For example: “persons with disabilities are ‘subjects’ and not ‘objects’—sentient beings like all others deserving equal respect and equal enjoyment of their rights.” (Quinn, 2010).

13 Again from the Committee on the Rights of Persons with Disabilities: “[Legal capacity] is the key to accessing meaningful participation in society.” (2014, para. 13).

14 Here a “capability” refers to a real opportunity—an ability in a much broader sense than a mental capacity.

15 How equal capability is achieved for people with profound mental impairments remains a further question. For discussion in the context of voting see: Wasserman & McMahan (2012).

16 Posing a related balancing question directly in response to the CRPD, in this journal issue Dawson suggests that involuntary treatment might in fact *promote* social inclusion: “Involuntary psychiatric treatment, for instance, could both limit a person's autonomy and promote their social inclusion, health, and standard of living. Would it therefore violate or promote the person's rights under the Convention as a whole?” (Dawson, 2015, p. XX).

17 For example, Nussbaum claims that, “Jury service is a recognized hallmark of fully equal citizenship, exclusion form which is stigmatizing … it is … a badge of civic equality.” (Nussbaum, 2009, p. 349).

18 The arguments for greater recognition of legal capacity in the sphere of personal decisions won't be examined in detail here as the aim of the paper is limited to exploring their applicability to questions of legal capacity in relation to criminal acts.

19 I note, however, that Bach and Kerzner have been critical of Nussbaum's approach to mental disability (Bach & Kerzner, 2010, pp. 69–72).

20 By way of contrast, a consequentialist account according to which holding others responsible serves only to influence behaviour, suggests that withholding responsibility on the basis of mental incapacity entails only that the person is unable to change (Duff, 1986, pp. 42-54; Smart, 1961). Given that we often attribute an inability to change to others, from a consequentialist perspective withholding responsibility does not seem to involve a withdrawal of recognition as a human being. Most contemporary theorists, however, hold that there is more to attributions of responsibility than just this consequentialist element.

21 Smith holds, therefore, that responsibility should be withheld only in the rarest of circumstances. She nonetheless suggests that having a “transient mental illness” could count as a circumstance where withholding responsibility is appropriate, on grounds that an act committed as a result of a delusional state, for example, cannot be said to reflect the person's judgements.

22 In this journal issue Slobogin argues that, “Compared to an acquittal “by reason of inanity,” an acquittal on lack-of-intent, self-defense of duress grounds is far less tarnishing.” (Slobogin, 2015, p. XX).

23 It may be the case that the mental requirements for criminal responsibility are in practice already minimal relative to standards for legal capacity in relation to personal decisions. As discussed later in the paper, this is the currently case in English law concerning children. In this journal issue, Hanna Pickard claims that this is also true of the current approach to personality disorder in English law (Pickard, 2015).

24 For example, Minkowitz writes, “Crises of self are not merely distressing; they can be valuable opportunities that push us to go beyond old assumptions and habits. In attempting to protect oneself or another person from risks we can inadvertently do more harm than good—not only the deprivation of opportunities for growth but also the harm done to the personality by such deprivation, which can lead to destructive consequences.” (Minkowitz, 2014, p. 2).

25 A trial of a supported decision-making in the state of South Australia provides some preliminary evidence for the possibilities in terms of personal growth as a result of a shift from guardianship to a supported decision-making structure (Wallace, 2012).

26 However, this intuition only goes so far. In the context of capacities for financial autonomy, for example, it is within the realms of pocket money and odd jobs that a young person's financial autonomy is enabled and developed. It would be against common sense to allow a 7-year-old to have a credit card with a standard limit. Richard Thaler and Cass Sunstein make a similar point when they agree that as a general principle, “it is sometimes helpful for us to make mistakes, for this is how we learn”, but that, “[we] do not believe that children should learn the dangers of pools by falling in a hoping for the best.” (Thaler & Sunstein, 2009, pp. 240-241).

27 It would seem that part of the purpose of censure is to engage with the person as a moral agent, in a developmental way. Thanks to Matt Matravers for this point.

28 In this journal issue Peay also discusses the balancing of these kinds of considerations (Peay, 2015).

29 Thanks to Jonathan Herring for making this point.

30 This concern seems to resonate with the position of the Committee on the Rights of Persons with Disabilities, when they state that the “functional approach [where a person's decision-making skills are considered to be deficient] is flawed for two key reasons. … The second is that it presumes to be able to accurately assess the inner-workings of the human mind” (2014, para. 13). Seemingly making a related point, Gerard Quinn speaks of “the enthronement of rationality as the touchstone of humanity” which “needs to be closely interrogated since most of us do not actually function rationally.” (Quinn, 2010, p.21).

31 For one interpretation of these arguments see: Craigie & Bortolotti (2014, 398-404).

32 This approach to framing the question is adapted from Allen Buchanan and Dan Brock's approach to framing the questions of competence in personal decisions (Buchanan & Brock, 1989).

33 Pickard very effectively makes this point using a hypothetical story about P: “P's story sharply highlights the tension in how people with personality disorder are treated at the hands of criminal and civil law because his mental state *at one and the same time* is deemed to meet the conditions required for criminal responsibility, and to warrant involuntary hospital admission. … There are no doubt many ways we might attempt to reconcile and rationalize P's treatment by criminal and civil law respectively. But it is nonetheless difficult not to feel, at heart, that P gets a raw deal. For, whichever way he turns, he is subjected to the strong arm of the law—deemed sufficiently mentally well to be punished for his crimes, but not deemed sufficiently mentally well to retain the right to make his own decisions about matters of serious importance to his own life, including whether or not to continue it.” (Pickard, 2015, p. XX; see also: Peay, 2011b).

34 Consent at age 16 and refusal at age 18.

35 In his analysis Lyons compares decisions that involve death as a consequence—life-ending treatment refusals and killings—in order to hold this element constant across the two contexts.

36 It is widely accepted in legal theory that criminal responsibility requires certain mental abilities (for an analysis of this issue see: Loughnan, 2012). The practice of not holding infants and people with mental disorders responsible is said to reveal a capacity requirement as a prerequisite for criminal responsibility, and according to Antony Duff this requirement is the capacity to be reasons responsive, at least to the relevant reasons (Duff, 1986). Being “reasons responsive” might be given as one account of what it is to be self-governing. However fleshing self-government out in this way exposes the possibility that there may be different relevant reasons in these two legal contexts. The relevant reasons may be “internal” reasons in the personal context, and “external” reasons in the criminal context, and this could be used to argue that there may be different psychological mechanisms in play (Craigie & Coram, 2013). This possibility is discussed briefly towards the end of this paper.

37 Lyons also cites a number of legal principles in support his presumption in favour of uniformity: a principle set out in the Beijing Rules according to which the burden of the criminal responsibility requires that the individual is able to claim correlative legal rights; and the United Nations Standard Minimum Rules of the Administration of Juvenile Justice (Lyons, 2010).

38 This assumption will be examined below.

39 Gerben Meyen has also argued for a more unified approach to legal capacity in these two contexts (Meynen, 2009, 2010).

40 I assume, as Lyons does, that the age threshold is at least in part justified in terms of mental abilities, with psychological development normally increasing with age.

41 I take this to be the point made by the Committee on the Rights of Persons with Disabilities when they write: “The concept of mental capacity … is not, as it is commonly presented, an objective, scientific and naturally occurring phenomenon. Mental capacity is contingent on social and political contexts, as are the disciplines, professions and practices which play a dominant role in assessing mental capacity.” (2014, para. 12).

42 Extreme examples would be assigning responsibility to a three-year-old, or not giving legal standing to adults on grounds of gender or race. Here the legal standards become completely detached from psychological considerations. The meaning of “responsibility” or “capacity” loses any connection with the psychological facts. In one possible example from existing medical law, Emma Cave has argued that the test for mental capacity applied in the English case of E (a woman refusing treatment for anorexia nervosa) was impossible to meet (Cave, 2014).

43 For example by ensuring that contracts were upheld.

44 For example, one judge held that “nothing ought to be more certain in the administration of the State's benevolence than that a sane person never passes behind the doors of its asylums as a prisoner.” *Van Deusen v. Newcomer,* 40 Mich. 90 (1897) at 137–8, as discussed in (Blumenthal, 2007, p. 1201).

45 The essential standard was described as the ability “to see things in their true relations, and to form correct conclusions” (*Dennett v Dennett,* 44 N.H. 531 (1863) at 538), and “to transact business with intelligence, and an intelligent understanding of what he was doing” (*Hovey v Chase*, 52 Me. 304, 315 (1863) at 316), as discussed in (Blumenthal, 2007, p. 1224).

46 Instrumental reasons were also given for this low threshold. It was argued that less “mind” is needed to execute a will because it is a topic that the person will have considered over their lifetime, and they will therefore already know their intentions, unlike making a decision about a new business proposition (Blumenthal, 2007, p. 1206-7; 1234).

47 If law concerning children is any indication, mental thresholds for criminal responsibility are already lower relative to standards for legal capacity in the context of personal decisions, making this an unlikely outcome. Our intuitions here will depend to a significant degree on evaluative features such as the perceived seriousness of the criminal act and the personal choice in question. The four broad approaches set out in this section assume that these other factors are being held constant across the two legal contexts.

48 An example of this kind of argument was discussed in the case study of 19th century American law in relation to the capacity to execute a will. See footnote 46.

49 I am indebted to the 2012–13 ‘Philosophy, Justice and Health’ graduate research seminar group, and the 2014-15 ‘Profound Impairment’ graduate research seminar group in the Department of Philosophy at University College London for their help in developing this point.

50 For example, see Pickard on the approach to personality disorder in English law (Pickard, 2015), and the situation for young people in English law as discussed above.

51 Lyons ultimately rejects this justification on the basis of the arguments discussed above.
